# Anxiety and Depression in Women with Polycystic Ovary Syndrome

**DOI:** 10.3390/medicina58070942

**Published:** 2022-07-16

**Authors:** Paweł Dybciak, Ewa Humeniuk, Dorota Raczkiewicz, Jan Krakowiak, Artur Wdowiak, Iwona Bojar

**Affiliations:** 1 Plastic Surgery and Advanced Laser & Skincare Aesthetics, “The Clinic” Warsaw, Krochmalna 59A Street, 00-864 Warsaw, Poland; paweldybciak@gmail.com; 2 Chair and Department of Psychology, Faculty of Medical Sciences, Medical University of Lublin, Chodzki 7 Street, 20-400 Lublin, Poland; ewahumeniuk@umlub.pl; 3 Department of Medical Statistics, School of Public Health, Centre of Postgraduate Medical Education, Kleczewska 61/63 Street, 01-826 Warsaw, Poland; dorota.bartosinska@gmail.com; 4 Department of Social Medicine, Medical University of Lodz, Żeligowskiego 7/9 Street, 90-752 Lodz, Poland; jankrakowiak@wp.pl; 5 Chair of Obstetrics and Gynecology, Faculty of Health Sciences, Medical University of Lublin, Staszica 4-6 Street, 20-081 Lublin, Poland; wdowiakartur@gmail.com; 6 Department of Women’s Health, Institute of Rural Health in Lublin, Jaczewskiego 2 Street, 20-090 Lublin, Poland

**Keywords:** polycystic ovary syndrome, anxiety, depression

## Abstract

*Background and Objectives*: Mental health disorders are often the consequence of hormonal disorders such as those accompanying polycystic ovary syndrome (PCOS), where changes in appearance and having to deal with a number of other problems occur due to this illness. The objective of this study was to determine the prevalence and severity of anxiety and depression symptoms, the level of ego-resiliency, and the ways that women with PCOS cope with stress compared to healthy women in order to determine the influence of socio-demographic characteristics in relation to levels of anxiety and depression with ego-resiliency and stress-coping methods. *Materials and Methods*: The study was conducted in Poland in 2021 and included 230 women with PCOS and 199 healthy controls aged 20–40 years old. The hospital anxiety and depression scale (HADs), ego-resiliency scale, as well as the MINI-COPE inventory were used. *Results*: The women with PCOS had higher levels of anxiety and depression and poorer ego-resiliency in comparison to the healthy women. The women with PCOS used passive stress-coping strategies significantly more commonly than the healthy women. Living in rural areas, having a lower level of education and being childless increased anxiety levels. Similarly, being over 30, living in a rural area, having a lower level of education, being childless, and being obese increased depression levels in the women with PCOS. A low level of ego-resiliency and passive stress-coping strategies are predictors of high levels of anxiety and depression in women with PCOS. *Conclusions*: Women with PCOS should be checked for anxiety and depression. They should also be checked to see whether they have the resources to cope with chronic stress in order to optimize therapeutic interventions.

## 1. Introduction

PCOS is the most common endocrine disorder. Its prevalence, depending on the diagnostic criteria employed, ranges from 2.2% to 26.7% worldwide [1]. According to the Rotterdam definition, which is the one most commonly used for PCOS, this disorder can be diagnosed in any woman who exhibits at least two of the following three symptoms: clinical and/or biochemical hyperandrogenism, ovulation dysfunction, or the presence of ovarian cysts in an ultrasound examination [2,3,4]. It is a heterogeneous illness, characterized by an unusually broad spectrum of clinical symptoms and involving many systems and organs [5]. The principal clinical symptoms include irregular periods or absence of menstrual cycle and/or infertility [6,7,8]; elevated insulin levels; insulin resistance and weight gain [9,10,11]; dyslipidemia and markers of endothelial dysfunction [12,13]; common acne, androgenic alopecia, and hirsutism [14,15]. These symptoms can affect a woman’s physical, social, and emotional well-being [4,16,17,18].

The occurrence of psychological disorders has been noted more commonly in women with PCOS [19,20]. In particular, depression and anxiety rates in women with PCOS have been well documented in the literature [4,21]. The most recent systematic review and metanalysis show that women with PCOS are many times more likely, in comparison to other women, to develop moderate to severe anxiety symptoms (as much as six times more likely) and depression symptoms (up to four times more likely) [4,19,22]. The prevalence of the clinically significant symptoms of depression in women with PCOS is 37%, compared to 14.2% in healthy women, and the prevalence of anxiety symptoms is 42%, compared to 8.5% in healthy women [20,23].

The mechanisms underlying the association of PCOS with anxiety and depression disorders are poorly understood [24,25]. They could be the result of the burdensome symptoms of PCOS, hormonal changes, or a combination of these factors. Increasing our understanding of the risk of mood disorders among women with PCOS could provide new therapeutic approaches. For this reason, the Androgen Excess-Polycystic Ovary Syndrome Society, in 2018, issued a call to action in which mental health in relation to PCOS was made a research priority [26].

Some studies associated the relationship between anxiety/depression disorders in women with PCOS and the ways in which women cope with stress, noting the personality trait known as ego-resilience [27,28]. Many studies suggest that high levels of stress are at the root of the mood and anxiety problems observed in women with PCOS [5,8,29,30,31]. Women with PCOS reported a significantly greater physiological reaction to stress in comparison to healthy women [8,16] and, moreover, were hospitalized twice as often due to stress and self-harming behavior [32]. High levels of stress have been shown to be associated, at least in part, with some clinical features of PCOS, including obesity, acne, hirsutism [33,34,35]; these also may be side effects of medication [21,36].

Stress-coping mechanisms are an important mediator in the relationship between the experience of stress and any mental disorders resulting from it [37]. There are two basic strategies for coping with stress [38]. Coping strategies are sets of individual cognitive and behavioral efforts that are used in order to change, adjust, and interpret a stressful situation and reduce suffering. Active coping (concentrating on the problem) is characterized by striving to resolve the problem. Passive coping (concentrating on emotions) is the use of strategies for reducing adverse emotions arising from a stressful situation [39].

Another study result suggested that passive coping may constitute a maladaptive strategy associated with anxiety and depression symptoms and leads to a worsening quality of life [40]. On the other hand, active coping may protect psychosocial well-being [41]. Studies by Benson et al., 2010; Sigmon et al., 2004, show that depression and anxiety are negative consequences of passive coping [16,42].

Until recently, it has not been common to analyze the coping strategies of women with PCOS [16,43,44,45]. Some studies indicate that women do use maladaptive coping processes [16,45]. Interestingly, some studies have found that most women with PCOS use adaptive stress-coping styles [43,44].

The next mediator for the relationship between stressful situations and their resulting mental disorders is the personality trait known as ego-resiliency. This is a characteristic that provides individuals with the necessary emotional, motivational, and cognitive resources for controlling their behavior and adapting to changing circumstances [46,47]. Ego-resiliency is considered a “meta-resource”, responding to stress with a flexible selection of coping strategies depending on the requirements of the specific difficult situation. Ego-resiliency reduces the tendency of experiencing anxiety and depression even when the given person perceives a certain circumstance as stressful [48]. Unfortunately, we were unable to find any studies on the topic of ego-resiliency in women with PCOS.

The aim of this study was to determine the prevalence and severity of anxiety and depression symptoms, the level of ego-resiliency, and the ways that women with PCOS cope with stress compared to healthy women and to determine any possible socio-demographic influence on these variables. A further aim was to evaluate the relationship of the levels of anxiety and depression with ego-resiliency and stress-coping methods in women with PCOS.

## 2. Materials and Methods

### 2.1. Study Group

The study was conducted in the gynecology-endocrinology clinics in the Lublin region in 2021 and included 230 women with PCOS, diagnosed according to the Rotterdam criteria [49]. To diagnose PCOS, two out of three criteria are required:

1. Increased serum concentration of androgens (hyperandrogenemia) or the presence of clinical signs related to androgen excess (hyperandrogenism);
2. Oligo- or anovulation;
3. Ultrasonographic-feature characteristics of polycystic ovaries.

The diagnosis was made after excluding other disorders causing similar symptoms.

A control group consisted of 199 age-matched women.

The author’s questionnaire was used to collect socio-demographic data, such as age, level of education, place of residence, type of employment, marital status, and the number of children, as well as weight and height to calculate body mass index.

Some psychological questionnaires were also used, and they are mentioned below.

Informed consent for the participation in the study was obtained from all the women.

The study was approved by the Ethics Committee of the Institute of Rural Medicine in Lublin, Poland.

### 2.2. Hospital Anxiety and Depression Scale (HADS)

Anxiety and depression symptoms were assessed with the hospital anxiety and depression scale (HADS) [50], under Polish translation [51]. The HADS is a self-report rating scale that consists of a total of 14 items divided into 2 subscales: anxiety and depression, with each subscale consisting of 7 items.

For each item, a 4-point response scale is used, and ranges from 0—“absence of symptoms” to 3—“maximum symptomatology”. The total score for each subscale is calculated as the sum of the 7 items. For each subscale, a respondent can obtain 0 to 21 points. The higher the score is, the higher the level of anxiety is. A score of 0–7 indicates no anxiety, a score of 8–10 indicates mild anxiety, a score of 11–14 indicates moderate anxiety, and finally a score of 15–21 indicates severe anxiety. Depression is scored in the same way as anxiety.

### 2.3. Ego-Resiliency Scale

Ego-resiliency was assessed with the ego-resiliency scale by Block and Kremen [52], adapted into Polish by Kaczmarek [53]. This scale consists of 14 items. For each item, a 4-point response scale is used: 1—“Does not apply at all”, 2—“Applies slightly”, 3—“Applies somewhat”, and 4—“Applies very strongly”. The total score of the scale is calculated as the sum of the 14 items. A respondent can obtain a score from 14– 56 points. The higher the score is, the higher the level of ego-resiliency is.

### 2.4. Coping Orientation to Problems Experiences Questionnaire (Mini-COPE)

Coping with stress was assessed with the MINI-COPE questionnaire by C.S. Carver et al. [54], adapted into Polish by Juczyński and Ogińska-Bulik [55]. This questionnaire consists of 28 items. For each item, there are four possible answers, as follows: “I haven’t been doing this at all” (scored as 0), “A little bit” (scored as 1), “A medium amount” (scored as 2), “I’ve been doing this a lot” (scored as 3).

Two items make up one strategy. In total, there are 14 stress-coping strategies, divided into four factors:

1. Active coping, which encompasses 3 strategies: Active coping, Planning, Positive reinterpretation;
2. Seeking support, which encompasses 2 strategies: Seeking emotional support, Seeking instrumental support;
3. Helplessness, which encompasses 3 strategies: Psychoactive substance use, Restraint, Self-blame;
4. Avoidant coping, which encompasses 3 strategies: Dealing with something else, Denial, Venting.

Moreover, there are three more strategies which do not belong to any of the previously mentioned factors and they constitute three separate factors: Religion, Acceptance, and Humor.

All strategies in all factors can be divided into active (effective) strategies and passive (ineffective) strategies. Active strategies constitute the following factors: Active coping, Seeking support, Religion, Acceptance, Humor. Passive strategies constitute the following factors: helplessness and avoidant coping.

Each strategy is scored as an arithmetic mean of the scores for the two appropriate items.

### 2.5. Statistical Methods

The statistical analyses were conducted using STATISTICA software. The mean (M) and standard deviation (SD) were estimated for the continuous variables, as well as the absolute numbers (n) and percentages (%) of the occurrence of the items for categorical variables.

The following statistical tests were used:

- Pearson’s chi-square test to compare the socio-demographic characteristics between the PCOS group and the control group, and the severity of anxiety and depression symptoms (measured as none, mild, moderate, or severe) between the PCOS group and the control group;
- Student’s *t*-test to compare ego-resiliency, the frequency of using stress-coping strategies, the severity of anxiety and depression symptoms (in scores) between the PCOS group and the control group, the severity of anxiety and depression within two levels of education, place of residence (urban and rural), single or married status, and presence of children;
- F test analysis of variance to compare ego-resiliency, and the severity of anxiety and depression (in scores) between three age groups, four types of employment, and four BMI groups.

The significance level was assumed to be 0.05 in all the statistical tests used.

## 3. Results

Table 1 presents the socio-demographic characteristics of the women with PCOS and the healthy women from the control group. These two groups of women did not significantly differ in respect to: age (*p* = 0.949), level of education (*p* = 0.132), place of residence (*p* = 0.873), type of employment (*p* = 0.094), marital status (*p* = 0.501), and presence of children (*p* = 0.344). Most women from both groups were aged 26–30 years, had tertiary level education, lived in urban areas, had a non-manual job, were single, and did not have children. However, the PCOS women had significantly higher BMI and significantly lower socioeconomic status than the women in the control group (*p* < 0.001 and *p* < 0.001).

**Figure 1:**
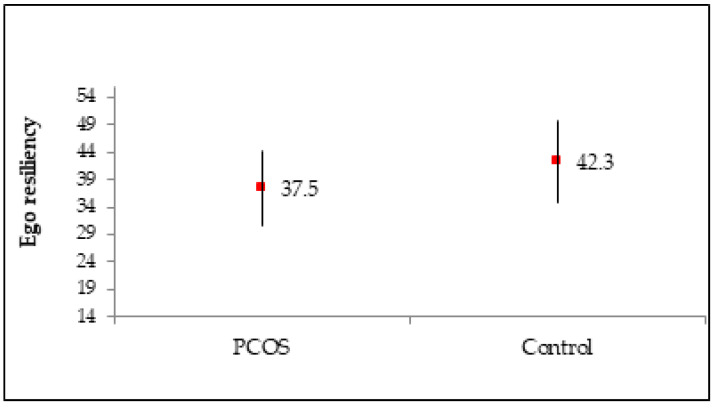
Ego-resiliency in the PCOS group and control group. Results are presented as mean ± standard deviation. Scale 14–56, mid-point = 35. *p* for Student’s *t* test.

The women with PCOS had significantly lower ego-resiliency (37.5, on average) than the healthy women from the control group (42.3, on average, *p* < 0.001, Figure 1). In the PCOS group, ego-resiliency did not correlate with age group (*p* = 0.715), level of education (*p* = 0.204), place of residence (*p* = 0.114), type of employment (*p* = 0.105), marital status (*p* = 0.862), having had children (*p* = 0.507), or BMI group (*p* = 0.908).

**Figure 2:**
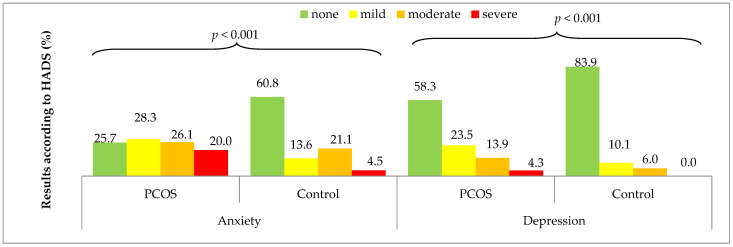
Severity of anxiety and depression symptoms acc. to HADS in the PCOS group and control group. Results are presented as *n* (%). *p* for chi-square test.

Figure 2 compares the severity of anxiety and depression symptoms (measured as none, mild, moderate, and severe) between the two analyzed groups. A significantly lower percentage of the PCOS women had no anxiety symptoms (25.7%) compared to the control group (60.8%, *p* < 0.001). The same differences referred to the severity of depression symptoms, which were significantly more common in the PCOS women (58.3% of them did not have depression symptoms) compared to the control group (83.9% of them did not have any, *p* < 0.001).

**Figure 3:**
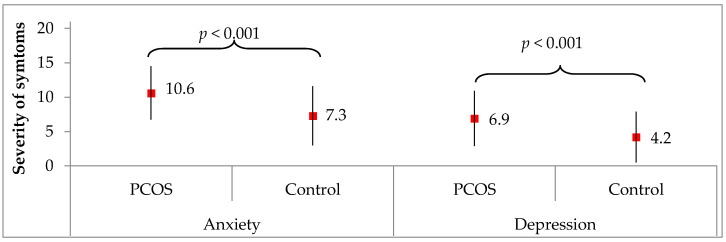
Severity of anxiety and depression symptoms acc. to HADS in the PCOS group and control group. Results are presented as mean ± standard deviation. *p* for Student’s *t* test. Scale 0–21: 0–7 none, 8–10 mild, 11–14 moderate, 15–21 severe.

Figure 3 compares the severity of anxiety and depression symptoms (in scores) between the two analyzed groups. The women with PCOS had a significantly higher severity of anxiety symptoms (10.6 on average) than the healthy women from the control group (7.3 on average, *p* < 0.001). The same differences referred to the severity of depression symptoms, which were higher in the PCOS women (6.9 on average) than in the control group (4.2 on average, *p* < 0.001).

**Figure 4:**
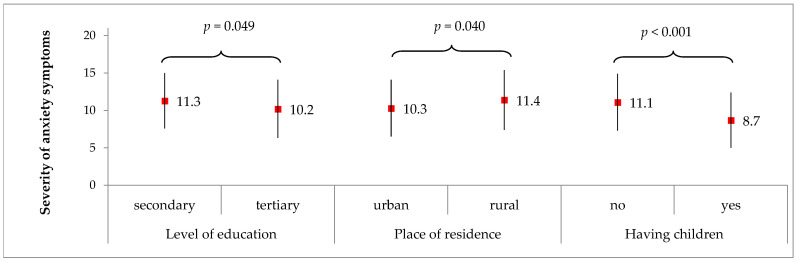
Significant correlations for the severity of anxiety symptoms acc. to HADS with socio-demographic characteristics in the PCOS group. Results are presented as mean ± standard deviation. *p* for Student’s *t* test.

Figure 4 presents significant correlations for the severity of anxiety symptoms with the socio-demographic characteristics in the PCOS group. In the women with PCOS, the severity of anxiety symptoms correlated significantly with the level of education (*p* = 0.049), place of residence (*p* = 0.040) and having had children (*p* < 0.001). The PCOS women had a significantly higher severity of anxiety symptoms if they had a secondary level of education compared to a tertiary level (11.3 vs. 10.2 scores, on average, respectively), lived in rural areas compared to urban areas (11.4 vs. 10.3, respectively) and had no children compared to the women who had children (11.1 vs. 8.7, respectively).

However, in the women with PCOS, the severity of anxiety symptoms did not correlate with age group (*p* = 0.104), type of employment (*p* = 0.178), marital status (*p* = 0.599), or BMI group (*p* = 0.649).

**Figure 5:**
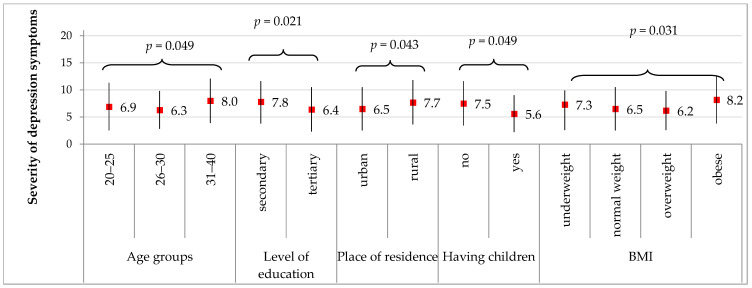
Significant correlations for the severity of depression symptoms acc. to HADS with socio-demographic characteristics in the PCOS group. Results are presented as mean ± standard deviation. *p* for Student’s *t* test or F test of analysis of variance. In the PCOS group, severity of depression symptoms did not correlate with *p* for Student’s *t* test for level of education, place of residence, marital status, and having had children. *p* for F test analysis of variance for age groups, BMI groups.

Figure 5 presents significant correlations for the severity of depression symptoms with socio-demographic characteristics in the PCOS group. In the women with PCOS, the severity of depression symptoms correlated significantly with age groups (*p* = 0.049), level of education (*p* = 0.021), place of residence (*p* = 0.043), having had children (*p* = 0.049), and BMI (*p* = 0.031). The PCOS women had a significantly higher severity of depression symptoms if they were over 30 years old compared to the 20–30-year-old women (8.0 vs. 6.9 and 6.3 scores, on average, respectively), had a secondary level of education compared to a tertiary level (7.8 vs. 6.4 scores, on average, respectively), lived in rural areas compared to urban areas (7.7 vs. 6.5, respectively), had no children compared to the women who had children (7.5 vs. 5.6, respectively), and were obese or underweight compared to normal weight or overweight (8.2 or 7.3 vs. 6.5 or 6.2, respectively).

However, in the women with PCOS, the severity of depression symptoms did not correlate with their type of employment (*p* = 0.119) or marital status (*p* = 0.255).

Table 2 compares the frequency with which different strategies to cope with distress were used between the women with PCOS and the healthy women. The women with PCOS more commonly used passive strategies (except for denial), they more often sought instrumental support, and used acceptance to cope with distress, compared to the healthy women (average scores were significantly higher for the PCOS women than for the healthy women). However, the women with PCOS less commonly used some of the active strategies (Active coping, Planning, Religion, and Humor) compared to the healthy women (average scores were significantly lower for the PCOS women than for the healthy women). However, the frequency of using these three strategies: Positive reinterpretation, Seeking emotional support, and Denial, did not significantly differ between the PCOS and the healthy women (*p* = 0.183, *p* = 0.605, and *p* = 0.345, respectively).

Table 3 presents the correlations for the severity of anxiety and depression symptoms with ego-resiliency and the frequency of using different strategies to cope with distress in the PCOS group. The lower the severity of anxiety and depression symptoms the women with PCOS had:

- the higher their ego-resiliency (r = −0.295, *p* < 0.001 and *r* = −0.499, *p* < 0.001, respectively);
- the more often they used some active strategies (Active coping, Planning, Positive reinterpretation, Seeking emotional support, and Humor) to cope with distress (r < 0, *p* < 0.05);
- the less often they used some passive strategies (Self-blame, Dealing with something else, Denial, and Venting) to cope with distress (r > 0, *p* < 0.05).

The severity of anxiety and depression symptoms in the women with PCOS did not correlate with the frequency of Seeking instrumental support, Acceptance, Psychoactive substance use and Restraint (*p* > 0.05). The more often the women with PCOS used Religion to cope with distress, the higher their severity of depression symptoms was (r = 0.180, *p* = 0.006), but the frequency of using Religion to cope with distress did not correlate with the severity of anxiety symptoms (r = −0.080, *p* = 0.226).

## 4. Discussion

It has been observed that mental health disorders are often the consequence both of hormonal disorders, such as those accompanying PCOS, and also unfavorable changes in appearance, as well as the necessity of dealing with a number of problems resulting from this chronic, untreatable illness. Two of the significant complications of PCOS, widely confirmed in previous scientific meta-analyses and systematic reviews, are anxiety and depression [20,29,56,57].

Although numerous studies have been conducted in this area, it has not yet been established what factors influence anxiety and depression levels in women with PCOS. For that reason, the aim of our study was not only to determine the frequency of these disorders but also to attempt to answer the question as to whether ego-resiliency level, stress-coping strategies, and socio-demographic variables have a significant influence on the level of anxiety and depression among women with PCOS. The study included 230 women with PCOS and 199 non-PCOS women as the control group, all of whom were in the reproductive age group of 20–40 years old. The studied group of women differed significantly in terms of BMI. Among the women with PCOS, exactly 50% were overweight or obese, while this problem was present in only 17% of the women from the control group.

Also, high levels of anxiety were confirmed in our research; anxiety was absent in only 25% of the examined women with PCOS, whereas 74.4% of the women reported mild (28%), moderate (26%), and severe (20%) anxiety symptoms. Over 60% of the control group confirmed that they had no anxiety, and only 4.5% reported severe anxiety. In a recently conducted study, only 6% of the women with PCOS had severe anxiety symptoms; the rest had mild or moderate symptoms [58].

The mean level of anxiety in the women we surveyed was 10.6, according to the HADS scale. It should be emphasized that compared to previous studies, this result is much higher: 10.1 [59], 9.0 [33], 8.8 [60].

The evaluation of the influence of socio-demographic factors showed that the severity of anxiety was significantly higher in the PCOS women living in rural areas with a lower level of education and in those who had no children compared to those living in the city with higher education and having had children. On the other hand, age, type of employment, marital status, and BMI had no bearing on the anxiety level in the women with PCOS.

There is evidence confirming a higher level of anxiety among childless women with PCOS [61,62]. However, no confirmation of the influence of the place of residence or education level was found in the literature, which could be an interesting discovery. Woman living in rural areas may have less accessibility or opportunity to get help in resolving their health problems. Moreover, they may be more exposed to pressure and negative judgments in a small community, which has a negative influence on their mental health [63]. In turn, more highly educated people, as the evidence indicates, are more effective at finding help and at coping with stress, which shows education to be a protective factor against anxiety symptoms [64].

The mean level of depression in our study was 6.9 and was significantly higher than in the healthy women (4.2). In comparison to the earlier observations, however, both the amount and the distribution of mild, moderate, and severe depression were similar [20,33,58,60,65].

It was found in our study that, in the women with PCOS, the severity of depression correlated with age, place of residence, level of education, having had children, and BMI. The link between the level of depression and childlessness has already been observed [20,58,62]. It is, however, important to note that the results of the other studies have not confirmed the existence of such a link [57,66]. A higher level of depression in the women with PCOS has been observed among overweight and obese women [4,56,60,67].

As in the case of anxiety, no confirmation of an influence regarding the place of residence or education level on depression has been found in the existing literature. In our study, a place of residence in a rural area and a lower level of education were predictors of a higher level of depression in the women with PCOS.

Another aim of our study was to assess the relationship between the character trait known as ego-resiliency, along with stress-coping strategies, with the severity of anxiety and depression symptoms in the women with PCOS.

In our study, the level of ego-resiliency in the women with PCOS was significantly lower (37.5 on average) than in the healthy women (42.3 on average) and showed no relationship to any of the controlled socio-demographic variables. It was found, however, that as the level of ego-resiliency increased, the level of anxiety and depression decreased on average. Since this was the first time that ego-resiliency has been considered a factor in the study of women with PCOS, it is not possible to compare these results. However, studies conducted on a group of women with high-risk pregnancies and a group of patients with rheumatoid arthritis have shown that a high level of ego-resiliency promotes life satisfaction and adaptation to illness [47,68]. Ego-resiliency can therefore be seen as a resource that not only promotes positive emotions but also supports a flexible selection of strategies for coping with the demands of a given situation [47].

Active stress-coping strategies, such as Active coping, Planning, Positive reinterpretation, Seeking instrumental support, Religion, and Humor were used more often by the healthy women, as opposed to passive strategies, which are often used by the women with PCOS. Strategies for coping with stress show a significant relationship with the level of anxiety or depression. The more active the strategy was, the lower the severity of anxiety and depression symptoms on average. Passive ways of coping with stress are associated with an increase in anxiety and depression. This can be explained by the fact that they have an acute, short-term effect, but, in the long run, they hinder psychological adaptation and aggravate symptoms of stress, medication, and depression [29].

In a German study, women more often used strategies concentrated on emotions, and using strategies focused on their problems did not have a significant influence on anxiety and depression [16]. A Turkish study, meanwhile, indicated that Self-blaming and Helplessness are the stress-coping strategies used most often by Turkish women with PCOS [69]. Signe (2021) demonstrated a link between coping strategies and anxiety and depression levels, and Kolahi (2015) showed a relationship between this and the quality of life of women with PCOS [45,70]. Some authors have found no difference between women with PCOS and healthy women in the way they deal with stress. Basirat (2020) even showed that women with PCOS have a higher level of Seeking support and Focusing on problems [44]. Coarron (2017) indicates that healthy women and those with PCOS are equally good at coping with difficult situations [43].

An assessment of coping strategies and ego-resiliency in women with PCOS can help to identify people who are at risk of a psychological well-being deterioration. It is also necessary to implement educational and therapeutic interventions to help women deal effectively with their illness. On the basis of other chronic diseases, it has been established that cognitive behavioral therapy can effectively modify coping strategies [71], thus contributing to better psychosocial adaptation to the illness and to greater mental well-being [72].

There were some limitations in our study, one being that it was cross-sectional in its nature, which limited the full assessment of all aspects of PCOS and its impact on the psyche of the patients. Another limitation was the use of self-reporting questionnaires, which could lead to biased responses. Full assessments of anxiety and depression were not possible due to the exclusive use of screening tools. Future research in this field should focus also on assessing the possibility of applying therapeutic interventions, both in the area of anxiety and depression and increasing the potential of the personality regarding flexible coping with the stress of a chronic illness.

## 5. Conclusions

The women with PCOS had higher levels of anxiety and depression and poorer ego-resiliency, and used passive stress-coping strategies significantly more commonly in comparison with the healthy women. Socio-demographic variables, such as living in rural areas with a lower level of education and being childless, increased anxiety levels. Being over 30 years old, living in a rural area with a lower level of education, being childless, and being obese increased the depression levels in the women with PCOS. A low level of ego-resiliency and the use of passive stress-coping strategies are predictors of high levels of anxiety and depression in women with PCOS. In accordance with the recommendations, and in order to optimize therapeutic interventions, women with PCOS should be checked for anxiety and depression as well as for their access to resources for coping with the stress of chronic illness.

## Figures and Tables

**Table 1:** Socio-demographic characteristics of PCOS group and control group.

Variable	Category	PCOS (N = 230)	Control (N = 199)	*p*
Age groups	20–25	86 (37.39)	75 (37.69)	0.949
	26–30	102 (44.35)	90 (45.23)	
	31–40	42 (18.26)	34 (17.09)	
Level of education	secondary	73 (31.74)	77 (38.69)	0.132
	tertiary	157 (68.26)	122 (61.31)	
Place of residence	urban	166 (72.17)	145 (72.86)	0.873
	rural	64 (27.83)	54 (27.14)	
Type of employment	student	48 (20.87)	48 (24.12)	0.094
	manual job	46 (20.00)	36 (18.09)	
	non-manual job	124 (53.91)	93 (46.73)	
	unemployed	12 (5.22)	22 (11.06)	
Socioeconomic status	very bad or bad	4 (1.74)	0 (0.00)	<0.001
	medium	59 (25.65)	51 (25.63)	
	good	143 (62.17)	93 (46.73)	
	very good	24 (10.43)	55 (27.64)	
Marital status	single	162 (70.43)	146 (73.37)	0.501
	married	68 (29.57)	53 (26.63)	
Have children	no	180 (78.26)	148 (74.37)	0.344
	yes	50 (21.74)	51 (25.63)	
BMI	underweight	6 (2.61)	15 (7.54)	<0.001
	normal weight	108 (46.96)	149 (74.87)	
	overweight	59 (25.65)	30 (15.08)	
	obese	57 (24.78)	5 (2.51)	

Results are presented as *n* (%). *p* for chi-square test.

**Table 2:** MINI-COPE inventory for PCOS group and control group.

	Factor	Strategy	PCOS (N = 230)	Control (N = 199)	*p*
Active strategies	Active coping	Active coping	1.6 ± 0.7	1.8 ± 0.6	0.001
		Planning	1.8 ± 0.6	2.0 ± 0.6	<0.001
		Positive reinterpretation	1.1 ± 0.7	1.0 ± 0.7	0.183
	Seeking support	Seeking emotional support	1.8 ± 0.7	1.8 ± 0.6	0.605
		Seeking instrumental support	1.7 ± 0.6	1.5 ± 0.7	0.001
		Religion	1.4 ± 0.7	1.8 ± 0.8	<0.001
		Acceptance	1.5 ± 0.6	1.3 ± 0.6	0.001
		Humor	0.7 ± 0.5	0.8 ± 0.6	0.051
Passive strategies	Helplessness	Psychoactive substance use	1.1 ± 0.6	0.7 ± 0.6	<0.001
		Restraint	1.6 ± 0.5	1.3 ± 0.5	<0.001
		Self-blame	1.9 ± 0.6	1.7 ± 0.6	0.002
	Avoidant coping	Dealing with something else	1.9 ± 0.7	1.7 ± 0.6	0.001
		Denial	1.3 ± 0.6	1.2 ± 0.5	0.347
		Venting	1.5 ± 0.7	1.3 ± 0.6	0.002

Results are presented as mean ± standard deviation. *p* for Student’s *t* test. 4-point scale: 0—“I haven’t been doing this at all”, 1—“A little bit”, 2—“A medium amount”, 3—“I’ve been doing this a lot”.

**Table 3:** Correlations of HADS with ego-resiliency and MINI-COPE inventory in the PCOS group.

Factor			Strategy	HADS-A		HADS-D	
				r	*p*	r	*p*
Ego resiliency				−0.295	<0.001	−0.499	<0.001
Active strategies	Active coping	Active coping		−0.403	<0.001	−0.247	<0.001
		Planning		−0.140	0.034	−0.179	0.006
		Positive reinterpretation		−0.471	<0.001	−0.506	<0.001
	Seeking support	Seeking emotional support		−0.209	0.001	−0.266	<0.001
		Seeking instrumental support		−0.018	0.784	−0.063	0.339
	Religion			−0.080	0.226	+0.180	0.006
	Acceptance			−0.028	0.672	−0.039	0.558
	Humor			−0.220	0.001	−0.183	0.005
Passive strategies	Helplessness	Psychoactive substance use		−0.045	0.495	−0.007	0.916
		Restraint		0.010	0.882	−0.020	0.769
		Self−blame		+0.160	0.015	+0.163	0.013
	Avoidant coping	Dealing with something else		+0.157	0.017	+0.244	<0.001
		Denial		+0.225	0.001	+0.137	0.039
		Venting		+0.133	0.043	+0.187	0.005

r—Pearson’s correlation coefficient.

## Data Availability

The datasets generated during the current study are available from Paweł Dybciak on reasonable request. The data are not publicly available due to privacy restrictions.
